# Lignin degradation potential and draft genome sequence of *Trametes trogii* S0301

**DOI:** 10.1186/s13068-019-1596-3

**Published:** 2019-10-30

**Authors:** Yuan Liu, Yuanyuan Wu, Yu Zhang, Xulei Yang, En Yang, Huini Xu, Qiliang Yang, Irbis Chagan, Xiuming Cui, Weimin Chen, Jinping Yan

**Affiliations:** 1 Faculty of Life Science and Technology, Kunming University of Science and Technology, Kunming, 650500 Yunnan China; 2 Biotechnology and Germplasm Resources Institute, Yunnan Academy of Agricultural Sciences, Kunming, 650223 Yunnan China; 3 Faculty of Modern Agricultural Engineering, Kunming University of Science and Technology, Kunming, 650500 Yunnan China; 4 Yunnan Provincial Key Laboratory of Panax notoginseng, Kunming, 650500 China; 5 Key Laboratory of Panax notoginseng Resources Sustainable Development and Utilization of State Administration of Traditional Chinese Medicine, Kunming, 650500 China; 6 Kunming Key Laboratory of Sustainable Development and Utilization of Famous-Region Drug, Kunming, 650500 China

**Keywords:** *Trametes trogii*, Genome sequencing, Lignin degradation, CAZymes, Laccases

## Abstract

**Background:**

*Trametes trogii* is a member of the white-rot fungi family, which has a unique ability to break down recalcitrant lignin polymers to CO_2_ and water, and they have enormous potential to biodegrade a wide range of toxic environmental pollutants. Because of its industrial potential, the identification of lignin-degrading enzyme systems in *Trametes* is an important area of research. Development and utilization of industrial value genes are suffering due to deficiency knowledge of genome available for their manipulation.

**Results:**

In the present study, Homokaryotic strains of *T. trogii* S0301 were screened and sequencing by PacBio Sequel II platform. The final draft genome is ~ 39.88 Mb, with a contig N50 size of 2.4 Mb, this was the first genome sequencing and assembly of *T. trogii* species. Further analyses predicted 14,508 protein-coding genes. Results showed that *T. trogii* S0301 contains 602 genes encoding CAZymes, include 211 glycoside hydrolase and 117 lignin-degrading family genes, nine laccases related genes. Small subunit ribosomal RNA gene (18S rRNA) sequencing confirms its phylogenetic position. Moreover, *T. trogii* S0301 has the largest number of cytochromes P450 (CYPs) superfamily genes compare to other fungi. All these results are consistent with enzymatic assays and transcriptome analysis results. We also analyzed other genome characteristics in the *T. trogii* S0301genome.

**Conclusion:**

Here, we present a nearly complete genome for *T. trogii* S0301, which will help elucidate the biosynthetic pathways of the lignin-degrading enzyme, advancing the discovery, characterization, and modification of novel enzymes from this genus. This genome sequence will provide a valuable reference for the investigation of lignin degradation in the *Trametes* genus.

## Background

Lignocellulosic biomass, consisting of cellulose, hemicellulose and lignin, is the most abundant renewable biomass and an enormous potential resource for bio-based industry [1]. White-rot fungi secrete a variety of intracellular and extracellular enzymes, including cellulases, hemicellulases, laccase, lignin peroxidases (LiP), and manganese peroxidases (MnP), to break down and utilize lignocellulose and naturally mineralize of the lignin [1, 2]. Thus, the genetic foundations and mechanisms of lignin degradation in white-rot fungi are major research focus areas for scientists in the field [1, 3].

Many species of white-rot fungi are gaining attention in both academic and industrial areas given their ligninolytic activities in nature [4, 5]. Due to the global spread of white-rot fungi in forests, *T. trogii* and other species in its genus have gained significant attention in both academic and industrial areas in recent years. *Trametes* genus is the model organism for wood degradation and lignocellulolytic enzyme production, particularly laccase [6–8]. *Trametes* is a major resource for waste treatment, due to its ability to degrade lignin and a broad range of diverse aromatic pollutants [3, 8–10]. The laccase production of multiple *Trametes* species has been studied, including *T. trogii*, *T. versicolor,* and *T. orientalis* [11–14].

To better understand the members of the *Trametes* genus, to date many nuclear genome sequences of *Trametes* species have been reported, including *T. versicolor* [15], *T. pubescens* [16], *Trametes* sp. AH28-2 [17], and *T. hirsuta* 072 [18] (Additional file 1). Many genes could be assigned to lignin-degrading enzyme systems. These results suggest that these species possess a complicated lignin metabolism system. However, genome sequencing of the above species has been based on first- and second-generation sequencing technology [10, 16–18], with a Contig N50 size range from 16,538 to 307,958 bp, except for *Trametes hirsuta* (Contig N50 = 3,045,029 bp) (Additional file 1). To date, the genome of *T. trogii* has not yet been reported.

*Trametes trogii* S0301 has multiple advantages. First, it is a thermotolerant strain with rapid growth and a growth optimum at 37 °C, higher than those of most white-rot fungi (28–30 °C) [6, 19–22]. Second, among enzymes involved in lignin degradation, laccase is predominant in *T. trogii* S0301, with the highest activity of 126 U/mL under submerged culture conditions [21, 22]. Third, it is well known that the characteristics of enzymes are usually optimized at the environmental temperature of the source organism [6]. The laccases from *T. trogii* S0301 are more thermotolerant than other laccases, with a maximum half-life of 3 h at 60 °C [21, 22]. Besides heat tolerance, the laccases from *T. trogii* S0301 have other advantages, including higher redox potential and higher tolerance to high metal ion concentrations. In summary, the advantages of *T. trogii* S0301 make it ideal for industrial applications to produce laccases with excellent properties using fast fermentation processes.

Therefore, the aim of the study was to obtain the genome sequence and high-quality annotations for *T. trogii* S0301, and clarify the enzyme characteristics mechanism in different glucose and lignocellulose culture medium. We also analyzed multiple genomic characteristics of the species, including repeat content, orthologous genes, CAZyme genes, and laccase pathway genes. The genome sequence of *T. trogii* S0301 will help researchers to realize the full potential of *T. trogii* as a source of ligninolytic activity and industrial enzymes.

## Results

### Genome sequencing, assembly, and annotation

After data filtering process by Trimmomatic v0.36, approximately 1.97 Gb (read length = 150 bp) and 6.87 Gb (read length = 150 bp) of clean data were obtained from the DNA and cDNA libraries, respectively. The study yielded 8.42 Gb of initially filtered PacBio data, consisting of 965,490 reads with 12.86 kb of subreads N50 (Additional file 2). The genome survey gave a size of 37.45 Mb and a heterozygous ratio of 1.17% based on ~ 50× Illumina sequencing reads (Additional file 3). Finally, PacBio reads gave ~ 220× genome coverage.

The de novo genome assembly resulted in a nearly complete genome of 39.88 Mb, with a contig N50 size of 2.4 Mb. The contig number was 29, the longest contig length was 4.82 Mb, and the shortest contig length was 20,367 bp. The GC content was 55.47% (Table 1).

**Table 1:** The de novo assembly result of *T. trogii* genome

Type	Value
Contigs number	29
Genome size	39,875,335 bp
Longest contig	4,822,965 bp
Shortest contig	20,367 bp
Contig N50	2,400,359 bp
Contig N90	1,007,640 bp
Number of sequences ≥ 3 kb	29
GC percentage (%)	55.47

Annotation results showed that the *T. trogii* S0301 genome contained 14,508 protein-coding genes with an average CDS length of 1258 bp. The percentage of spliced genes was 92.2%. The average protein sequence length was 419 bp, and the number of introns per gene was 4 (Additional files 4, 5).

A BUSCO evaluation showed that the *T. trogii* S0301 in genome and protein mode were similarly conserved, match 91.2% and 90.8% of complete BUSCOs, respectively (Additional file 6). Compare to other eight sequenced *Trametes* species, the complete BUSCOs match above 90%, except *T. villosa* (Additional file 7). We obtained 82.04 Gb RNA-seq clean reads from 10 samples, the average mapping rate is 96.59%. All 14,508 genes were annotated by RNA-seq data, and 731 new genes were found in the transcript annotation result. In the present assembly genome, 95.20% (14,508/15,239) genes were found (Additional file 8). The number of *T. trogii* S0301 protein-coding genes is similar to those of *T. hirsuta* (14,598), *T. pubescens* (14,718) and *T. versicolor* (14,572), but larger than *T. coccinea* (12,693) and *T. cinnabarina* (10,441) (Additional file 1). In total, the 14,508 protein-coding genes in *T. trogii* S0301 were comprised of 5833 multiple-copy orthologs and 2753 single-copy orthologs, therefore, a total of 8586 gene families were identified. Additionally, there were 3129 unclassified genes.

**Fig. 1:**
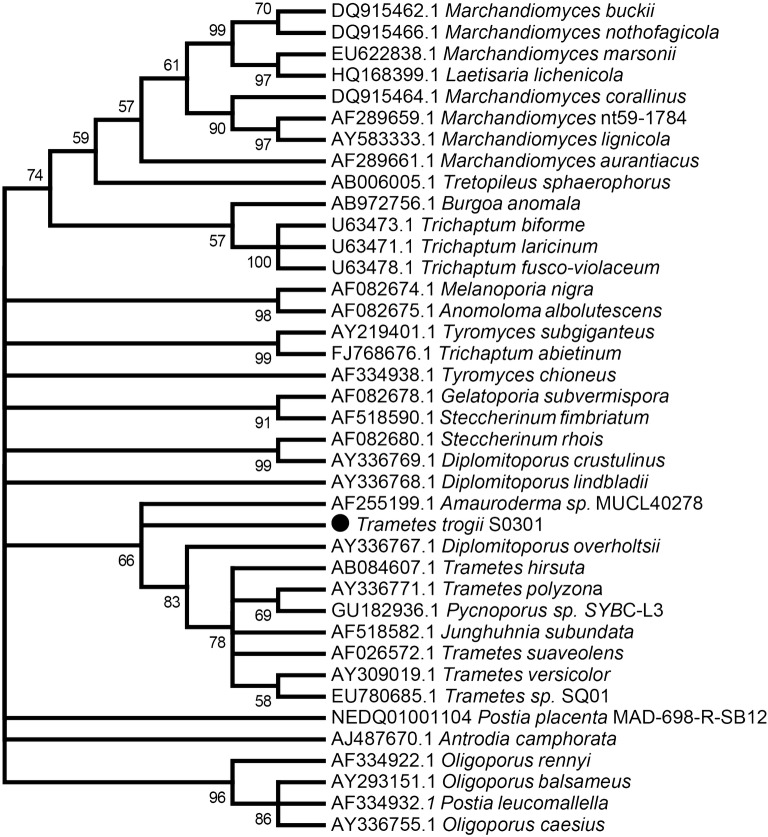
Maximum likelihood phylogenetic tree from 18S rRNA gene sequences of *T. trogii* S0301 and related species

The trametoid clade includes most *Trametes* species, including *T. suaveolens*, *T. versicolor*, and the mainly tropical species such as *T. maxima* and *T. cubensis*. It also includes species of the genera *Lenzites* and *Pycnoporus* and *Coriolopsis polyzona* [23]. The phylogenetic tree from 18S rRNA sequences grouped *T. trogii* with *T. polyzona*, *T. hirsuta*, *T. suaveolens* and *T. versicolor*. *T. trogii* is the basal species of the *Trametes* genus, which is close to *Amauroderma* sp. MUCL 40278 (Fig. 1). Our phylogenomic results are consistent with the consensus taxonomic status of *T. trogii* [23].

434 retroelements were found in the *T. trogii* S0301 genome, the majority of which belonged to the LTR (long terminal repeat) family (Gypsy/DIRS1 = 228, Ty1/Copia = 193) and 13 LINEs (long interspersed nuclear elements). SINEs (short interspersed elements) and Penelope elements were not detected. There were also 70 DNA transposons and 10 unclassified repeats in the genome. In total, 458,794 bp of the *T. trogii* S0301genome sequence belongs to interspersed repeats, corresponding to 1.15% of the entire genome. There were 26 small RNA, 5767 simple repeats, and 916 low-complexity repeats, corresponding to 0.85% of the genome (Additional file 9). Combining existing genomics results with the results in this paper, genome assembly and annotation of *T. trogii* S0301 achieved higher-quality.

### Gene function

**Fig. 2:**
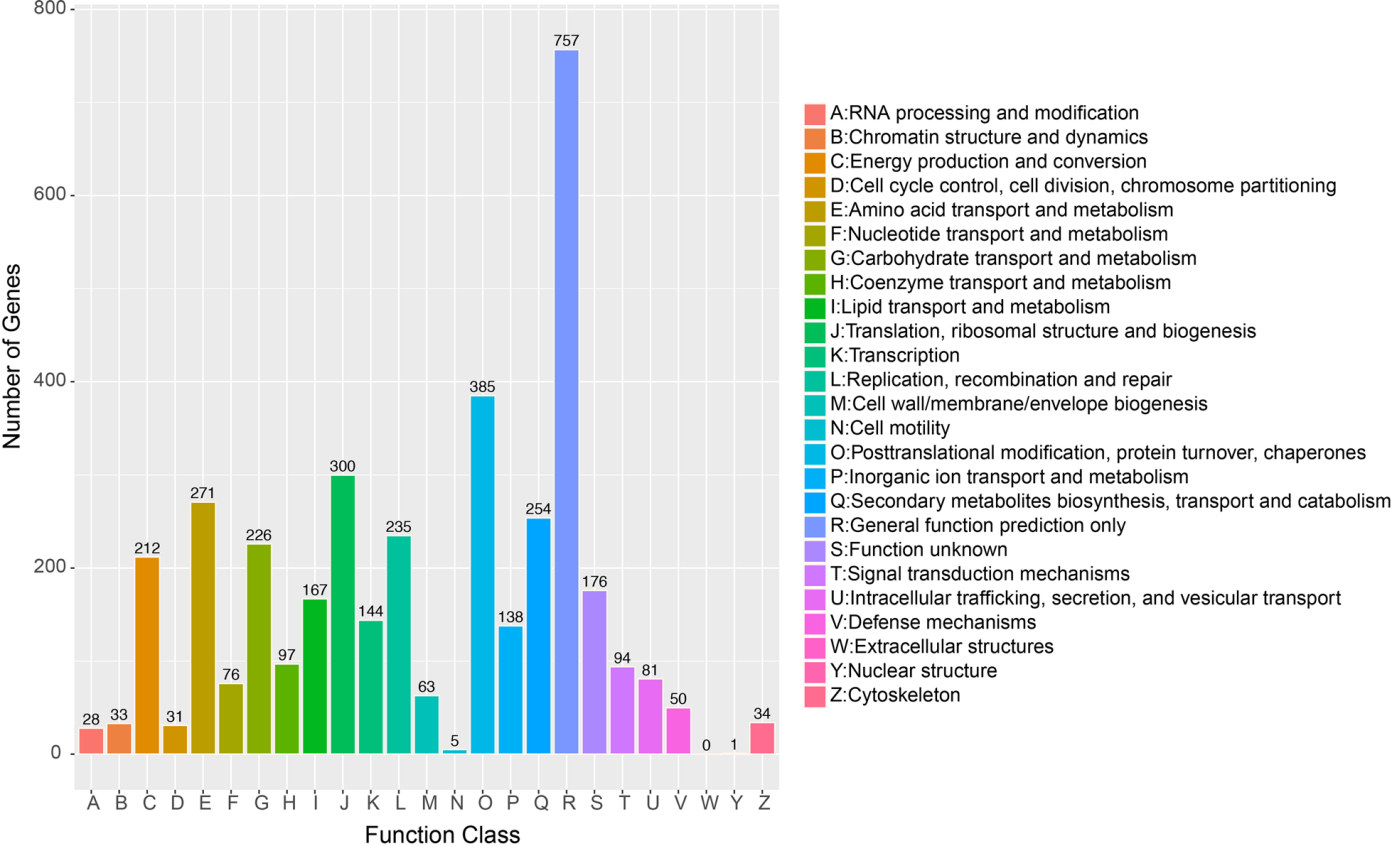
Clusters of Orthologous Groups of proteins (COG) functional classification of *T. trogii* S0301 genes

The Clusters of Orthologous Groups (COG) annotation results showed that 4779 (33%) genes were annotated in 1439 COG categories (Fig. 2). “General function prediction only” functional categories contained the largest number of genes. The most gene-rich classes in the COG function class were “posttranslational modification, protein turnover, chaperones (385),” “translation, ribosomal structure and biogenesis (300)” and “amino acid transport and metabolism (271).”

Carbohydrate transport and metabolism function are essential in lignin degradation, which can catalyze the transport of multiple substrates including ions, carbohydrates, lipids, amino acids, peptides, nucleosides, and other small molecules [24]. There were 226 genes were assigned to the “carbohydrate transport and metabolism” function class. Studies have shown that lignin peroxidase is an extracellular enzyme that breaks down lignocellulose [1]. Our data showed 81 genes in the “intracellular trafficking, secretion, and vesicular transport” COG function class, which are important for secretion of extracellular enzymes (Fig. 2).

**Fig. 3:**
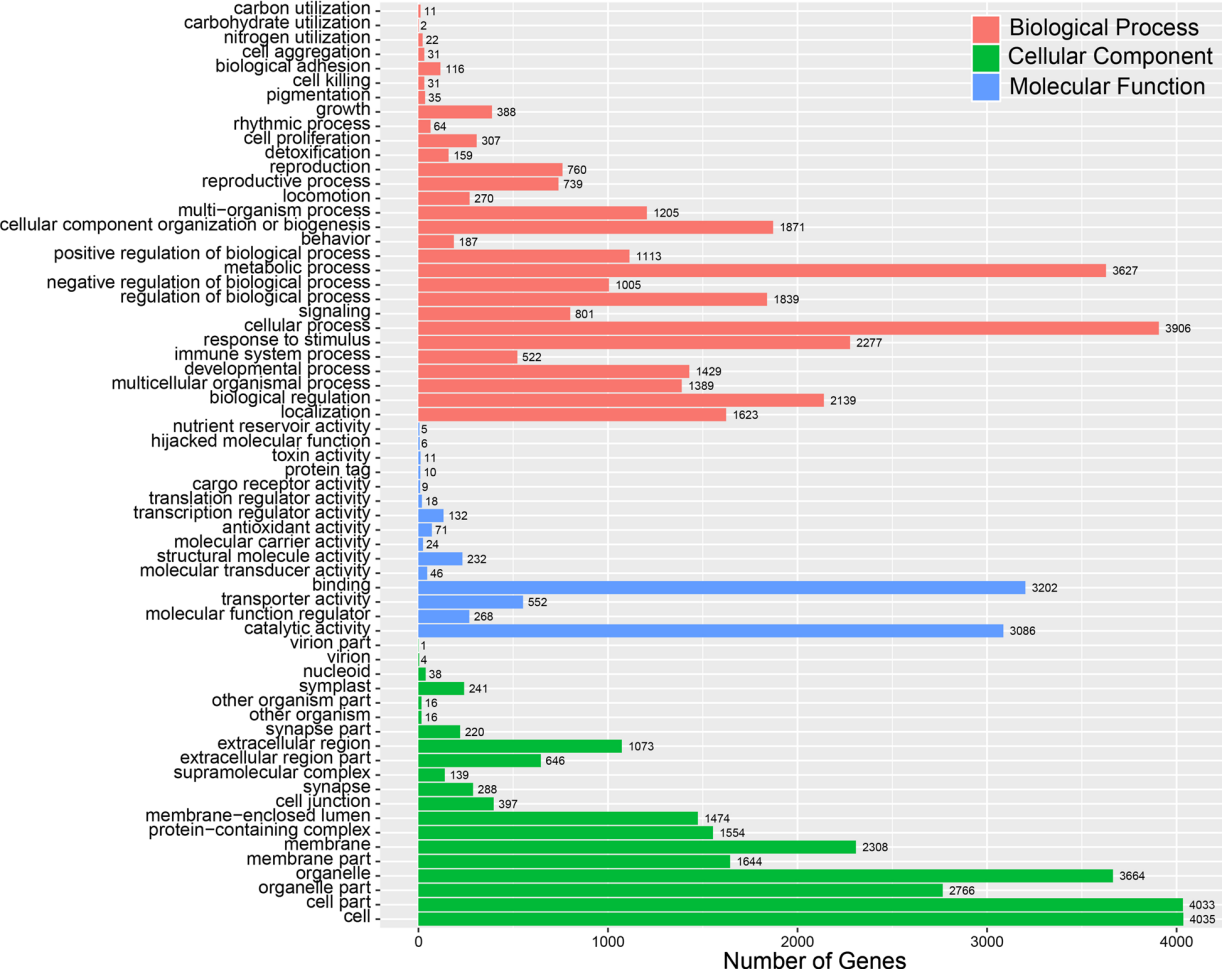
Gene Ontology (GO) functional annotations for *T. trogii* S0301

According to the Gene Ontology (GO) annotation results, 4559 genes were assigned to the GO database, accounting for 31% of the annotated genes, with most having multiple GO terms (Fig. 3). Genes were categorized by biological process, cellular component, and molecular function categories. The cellular component class was largest, followed by the biological process and molecular function classes. The classes relevant to lignin degradation contain large numbers of genes: cellular process (GO:0009987, 86%), metabolic process (GO:0008152, 80%), and catalytic activity (GO:0003824, 68%). Lignin-degrading white-rot fungi have the unique ability to degrade a broad spectrum of structurally diverse environmental pollutants [25], including polychlorinated biphenyls and detoxification of dyes and wastewaters [26, 27]. In *T. trogii* S0301 genome, 170 genes were found related to toxin activity (GO:0090729, 0.2%) and detoxification (GO:0098754, 3.5%) sub-functions.

**Fig. 4:**
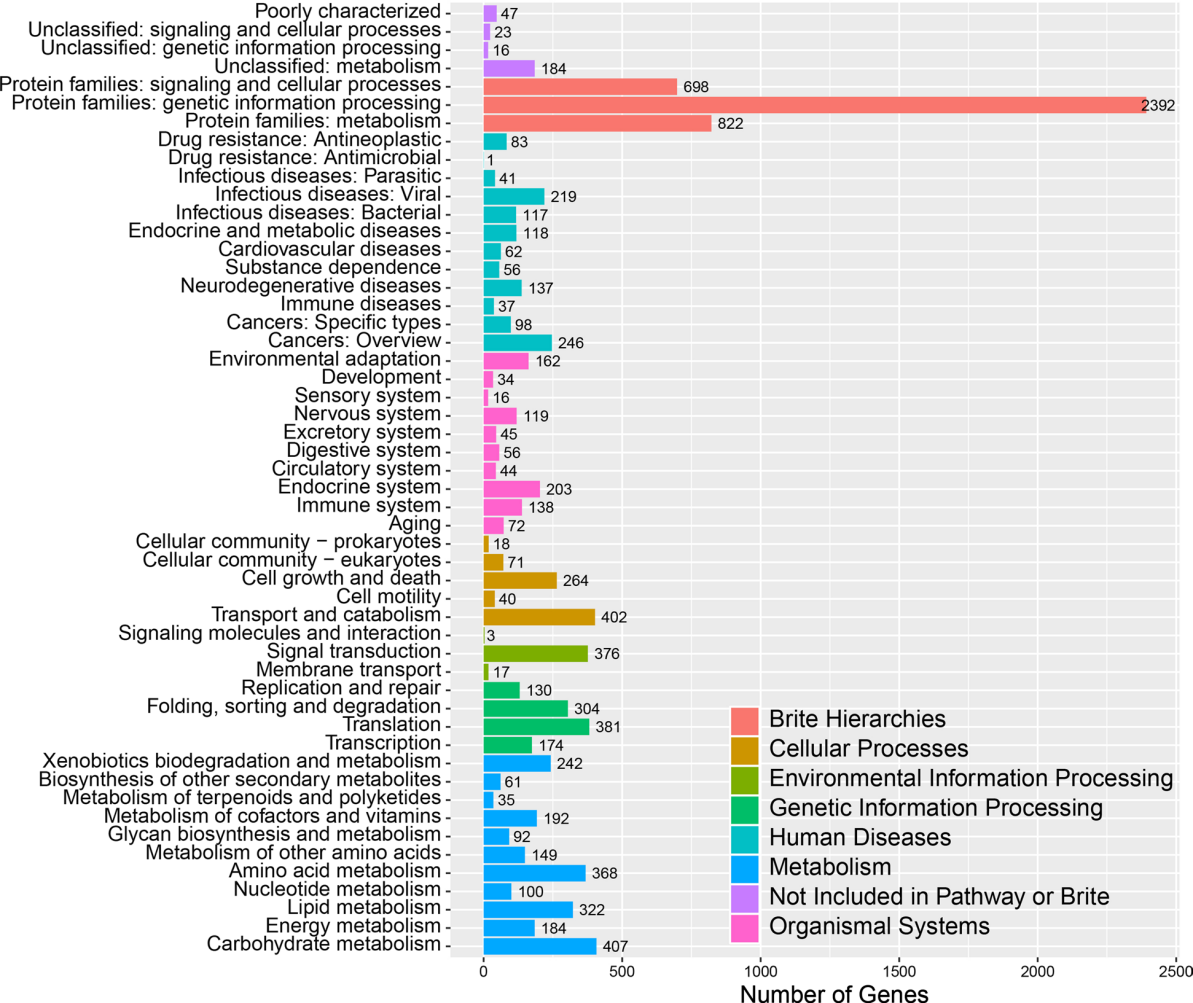
Kyoto Encyclopedia of Genes and Genomes (KEGG) functional annotations for *T. trogii* S0301

To further understand gene function in *T. trogii* S0301, 4322 putative proteins were successfully assigned to their orthologs in the Kyoto Encyclopedia of Genes and Genomes (KEGG) database (Fig. 4). Proteins assigned to 11 metabolism categories in KEGG were highly enriched, including “carbohydrate metabolism,” “amino acid metabolism,” “lipid metabolism,” “metabolism of terpenoids and polyketides,” and “glycan biosynthesis and metabolism.” Carbohydrate metabolism has special relevance in mycorrhizal fungi because they take up and utilize carbon photosynthates donated by plants [28]. “Membrane transport” and “endocrine system” may be related to the secretion of extracellular enzymes. “Transport and catabolism” and “xenobiotics biodegradation and metabolism” may be related to lignin degradation and detoxification activity.

In fungi, cytochrome P450 (CYPs) superfamily play diverse and critical roles in metabolism and fungal adaptation to specific ecological niches [29]. A total of 158 CYP genes were identified in the *T. trogii* S0301 genome. The functional category mainly distributed in “Lipid metabolism.” *T. trogii* S0301 has the largest number of CYP genes compare to other fungi, such as *Aspergillus flavus* (153) belong to Ascomycota, *Postia placenta* (106) belong to Basidiomycota, *Rhizopus oryzae* (49) belong to Zygomycota, and *Batrachochytrium dendrobatidis* (9) belong to Chytridiomycota [29].

These findings are suggestive of the presence of an enriched and varied array of metabolic functions that enable better absorption and transformation of material from substrates. The diversity of gene function suggests a high potential for *T. trogii* S0301 for environmental lignin degradation and adaptation.

### Analysis of CAZymes gene characteristics

**Fig. 5:**
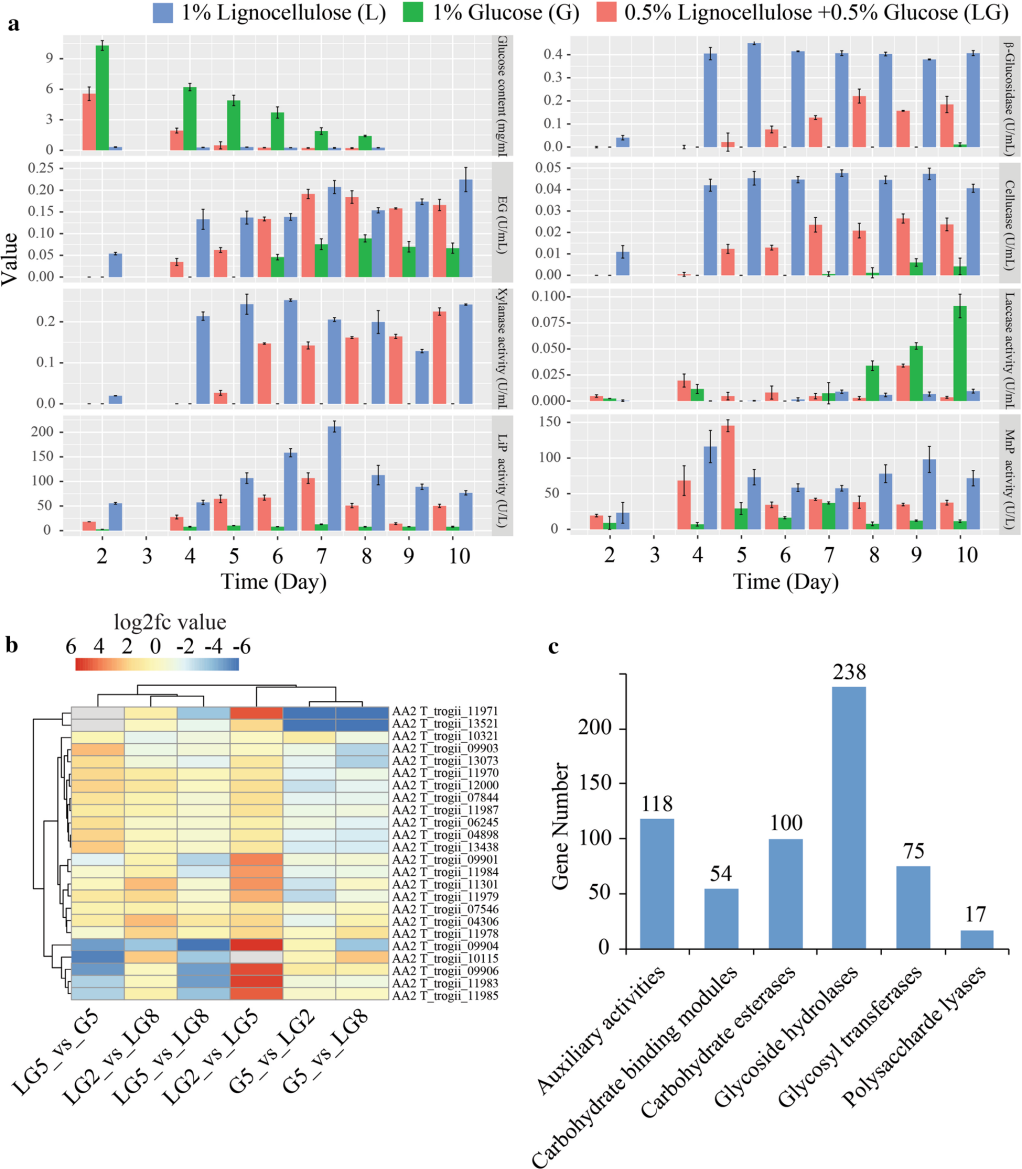
Time difference biochemical analyses of the lignocellulose-degrading enzyme activities of *T. trogii* S0301. **a** Different enzymes activity in 1% lignocellulose (L), 1% glucose (G), and 0.5% lignocellulose + 0.5% glucose medium (0.5% LG); **b** AA2 family gene expression profiles for the lignin-oxidizing enzymes associated with lignin modification under six culturing conditions; **c** distribution of CAZyme gene families of *T. trogii* S0301

The plant cell wall primarily consists of cellulose, hemicellulose and pectin. The presence of lignin along with these components make the plant cell wall recalcitrant. Fungi secrete an array of CAZymes and lignin-degrading enzymes (including aromatic compound-degrading and detoxifying enzymes) for the degradation of lignocellulose [30]. The total encoding CAZymes gene number was 602, in which glycoside hydrolases (GHs), auxiliary activities (AAs), carbohydrate esterases (CEs), glycosyltransferases (GTs), carbohydrate-binding modules (CBMs ), and PLs (polysaccharide lyases) accounted for 39.53%, 19.60%, 16.61%, 12.46%, 8.97%, and 2.82% (17), respectively (Fig. 5c and Additional file 10).

The fungal oxidative lignin enzymes database has classified lignin-degrading enzymes into lignin-oxidizing enzymes (LO) and lignin-degrading auxiliary enzymes (LD) [1]. In the CAZymes database, lignin-degrading enzymes are subdivided into the AA class; LO into the AA1, AA2, AA3 classes; and LD into the AA4, AA5, AA6 and AA8 classes. Our results showed that *T. trogii* S0301 possesses a large number of lignin-degrading enzyme genes (118/602, 19.60%). AA1 class enzymes (laccase) harbor nine genes. AA2 class enzymes (LiP, MnP and versatile peroxidases) harbor 24 genes. AA3 class enzymes (glucose methanol choline oxidoreductases) includes 30 genes within AA3_2 class enzymes (aryl alcohol oxidase and glucose-1-oxidase) harbor 17 genes. In most of the white- and brown-rot fungi, genes encoding vanillyl alcohol oxidase (AA4) are reduced to 0, while other fungi have 1–3 such genes [31]. Interestingly, two genes belong to AA4 class were found in the *T. trogii* S0301 genome. The number of genes encoding lytic polysaccharide monooxygenases (LPMO) are 20 with 1 in the AA9 and AA11 classes, respectively, while neither the AA10 nor AA13 classes had representatives in the *T. trogii* S0301 genome. Beside lignin-degrading enzymes, there are many genes encoding degradative enzymes, including those that degrade cellulose, hemicellulose, and pectin (Additional file 10). Relative to the CAZymes in white-rot, brown-rot, and soft-rot fungi [31], The results show that the total number of genes encoding lignin-degrading enzymes is higher than soft-rot fungi (13–115), brown-rot fungi (21–53), and *T. versicolor* (89) [31].

Laccase (EC1.10.3.2) is a group of copper-containing polyphenol oxidases which belongs to multi-copper oxidases family [32]. Laccase was used in pulp and paper industries, textile and dye industries. They also are exploited for bioremediation, organic synthesis, nanobiotechnology, cosmetics, cross-linking of polysaccharides, medical applications, enzymatic assays, and immunochemical assays [22]. White-rot fungi are the major producers [33]. Our previous research suggests that the laccase isoform of *T. trogii* S0301 strain has the potential for dye decolorization at high temperatures and high ionic concentrations [21, 22]. Nine genes encoding enzymes of the laccase pathway are present in *T. trogii* S0301 genome, consistent with the CAZymes and functional annotation data (Additional files 10, 11). Numerous laccase genes encode protein isoforms, including the N-terminal secretion signal peptide of 20 amino acids [34]. This is significant because signal peptides can be replaced by the *α*-factor prepro-leader to enhance the activity of laccases [35]. The signal peptide analysis showed that 7 genes contained signal peptides, 17–24 residues in length (Additional file 11).

### Lignocellulose-degrading enzyme activities of *T. trogii* S0301

For enzymatic assays, *T. trogii* S0301 was employed to explore the responses of the lignocellulose‑degrading enzymes and their encoding genes to lignocellulosic substrates. *T. trogii* S0301 is typically grown in Highley’s basal salt medium adding 1% glucose or 1% lignocellulose as carbon source. About 47.6% glucose was utilized on day 5, and the glucose consumption was almost completed on day 9 (Fig. 5a).

Enzyme activities including β-glucosidase, endoglucanase (EG), cellulase, xylanase, LiP, and MnP are significantly increased in 1% lignocellulose medium, while the activity of this enzyme, with the exception of EG and laccase, in medium with 1% glucose as a carbon source was undetectable. During the incubation period, LiP and MnP activities in 1% lignocellulose increased gradually, and reached their highest values of 211.6 U/L on day 7 and 116.1 U/L on day 4, respectively. After reaching their maximum, the level of LiP and MnP activities fell to about 70 U/L on day 10 (Fig. 5a). β-glucosidase, EG, cellulase, and xylanase activities in 1% lignocellulose medium showed a synchronous increase and reached to their maximum enzyme activities of 0.450 U/mL, 0.207 U/mL, 0.048 U/mL, 0.253 U/mL on day 5, day 7, day 7, and day 6, respectively.

To further explore the responses of lignocellulose‑degrading enzymes to lignocellulose substrates, we monitored the dynamic changes of glucose concentration and the activity in the lignocellulose degrading enzymes of *T. trogii* S0301 in 0.5% lignocellulose and 0.5% glucose (LG) culture. With the growth of mycelium, the concentration of glucose in 0.5% LG decreased gradually. The consumption of glucose reached 65% on day 4, and almost completed on day 5. At the same time of glucose depletion, lignin-degrading enzymes such as β-glucosidase and EG increased simultaneously with MnP, LiP, cellulase and xylanase activity, which were consistent with the results of single lignocellulose as carbon source (Fig. 5a).

### Expression of lignocellulose-degrading enzymes based on transcriptomic analysis

Based on the enzymatic assay results, total RNAs were extracted from the mycelia of *T. trogii* S0301 cultured in media containing 1% glucose, 0.5% lignocellulose + 0.5% glucose on day 5 and day 2, 5, 8 respectively. The up-regulation percentage of glycoside hydrolase encoding genes was significantly in LG (0.5% lignocellulose + 0.5% glucose) on day 2, day 5, and day 8. Interestingly, comparing the transcriptome of LG5 (LG on day 5) and G5 (%1 glucose, on day 5), a lot of CAZymes encoding genes show a down-regulation trend, may be related to the fact that the *T. trogii* S0301 uses glucose first and then lignocellulose (Fig. 5a and Additional file 12). Comparing the transcriptome of G5 with LG8, G5 with LG2, and LG5 with LG8 by GO analysis, the differently expressed genes were enriched in hydrolase activity and carbohydrate binding. The phenomenon is consistent with the result of CAZymes gene annotation (Fig. 5c). We also analyzed the differently express genes of AA2 family, comparing the transcriptome of LG2_vs_LG5 and LG5_vs_LG8, all 22 genes show up-regulation except T_trogii_10321 and T_trogii_09903 show down-regulation, consistent with the enzymatic assays results that the LiP and MnP activity significant increase on day 5. Subsequently, the LiP and MnP activity decreases on day 8 (Fig. 5a, b).

## Discussion

White-rot fungi have been known to possess the ability to break down the recalcitrant lignin polymer to carbon dioxide and water. These fungi also have the potential to biodegrade a wide range of toxic environmental pollutants [17, 36]. Until now, there are eight sequenced genomes of *Trametes* genus, and most of them have the ability to biodegrade lignocellulose and xenobiotics [37], but does not include the *T. trogii* species. Compare to other *Trametes* species, *T. trogii* S0301 has multiple advantages, such as thermotolerant high temperature [6], highest laccase activity [21, 22]. In this study, we sequence and de novo assembly *T. trogii* S0301 genome by PacBio sequencing technology, we found that the genome of *T. trogii* S0301 genome is 39.88 Mb, slightly larger than that of formerly chromosome level genome of *T. hirsuta* (37.43 Mb), and have a long enough contig N50 size. Systematic analysis of the CAZymes encoding gene characteristics of *T. trogii* S0301 genome, combined with the enzymatic assays and transcriptomic analysis showed that *T. trogii* S0301 contains 602 CAZyme encoding genes. In addition, the total number of genes encoding lignin-degrading enzymes is higher than soft-rot fungi (13–115), brown-rot fungi (21–53), and *T. versicolor* (89) [31]. The 18S rRNA phylogenetic analysis suggested that *T. trogii* S0301 is very closest to *Amauroderma* sp. MUCL40278 and *T. trogii* S0301 is the basal species of *Trametes* genus (Fig. 1). Whit-rot fungi are the main producers of ligninases that substantially contribute to lignin decay of wood [15, 38], compare to other fungi, *T. trogii* S0301 genome sequence and transcriptomic analysis revealed a large amount of CAZymes and CYPs genes involved in lignin and lipid metabolism. The sequenced and annotated genome of *T. trogii* S0301 presented here provides an excellent platform for subsequent biochemical and transcriptomic analyses of this fungus grown on lignocellulose and glucose.

Fungi produce a wide range of extracellular enzymes to break down plant cell walls, which are composed mainly of cellulose, lignin and hemicellulose. Among them are the GHs [39]. GHs are the most diverse group of enzymes used by microbes in the degradation of biomass. A lot of GHs families have been classified to date [39]. Many of them are responsible for the hydrolysis of the carbon–oxygen–carbon bonds that link the sugar residues in cellulose and hemicelluloses [39–41]. Compare to 1% glucose as carbon source, enzyme activities including β-glucosidase, EG, cellulase, xylanase, LiP, and MnP are significantly increased in 1% lignocellulose medium (Fig. 5a). A lot of CAZymes encoding genes are highly expressed at day 5 and day 8 in 0.5% LG medium, most of belong to the GHs family (Additional file 12).

*Trametes trogii* S0301 produces LiP, MnP and Laccase [21] whereas other lignin decay fungi produce only one or two of these ligninolytic enzymes simultaneously [42]. Laccases have been found in many fungi, including non-ligninolytic members of the Ascomycota, such as *Aspergillus* and *Neurospora*, as well as wood-rotting Basidiomycota. Laccases also occur in plants where they contribute to lignin biosynthesis. In this study, we found nine laccase pathway-related genes which belong to AA1_1 and AA1_2 family, eight genes have expression value in three different mediums, except T_trogii_01688.t1 (Additional file 11). MnP, LiP and laccase enzyme are the main lignin-degrading enzymes [43]. In 1% lignocellulose medium, MnP and LiP have high activity, and laccase activity very few in *T. trogii* S0301. This phenomenon is similar to *Phanerochate chrysosporium* and different from *T. versicolor* [1, 43] (Fig. 5a). Unlike *P. chrysosporium* lacking the gene that encoded for laccase [1], however, *T. trogii* S0301 possessed nine laccase isoenzymes encoding genes (Additional file 11) in its genome and showed high laccase-producing ability under the submerged culture conditions on GYP medium containing 2 mM CuSO_4_ in our previous studies [21]. Differences in components in the medium, culture conditions and stage could be factors in these differences [44], and the concentration of Cu^2+^ in the medium, a key inducer of laccase expression and activity, may be the main reason in this study that laccase activity has a very lower value on different medium even at the late stage (Fig. 5a).

White-rot fungi are a heterogeneous group that may degrade greater or lesser amounts of cell wall component. Some species preferentially remove lignin from wood, leaving pockets of white degraded cells that consist entirely of cellulose. This is defined as selective delignification. Other species degrade lignin and cellulose simultaneously which is defined as nonselective delignification [45, 46]. Combined the enzymatic assay and transcriptome result showed that *T. trogii* S0301 degrades lignocellulose non-selectively, same as *T. versicolor* [45].

In summary, the nearly complete genome of the *T. trogii* S0301 makes it a compelling model for studying the lignin catabolism of *Trametes* fungi. The identification of numerous lignin degradation enzymes will accelerate the discovery of complete lignin degradation mechanism for the strategic exploitation of these enzymes in industrial settings and will pave the way for its future roles in ligninolytic and industrial enzymes application.

## Conclusion

This is the first de novo assembly and annotation of a *T. trogii* genome. This represents an important resource for the species and is an improvement on the previous first- and second-generation sequencing of the *Trametes* genus. The nearly complete genome and high-quality annotation will provide a valuable genome resource for the investigation of lignin degradation by fungi of the *Trametes* genus. All of these genes encoding CAZymes can be used for breeding of improved strains in the future, enhancing ligninolytic activity and industrial enzymes application value of *T. trogii* species.

## Materials and methods

### Sample preparation, library construction, and sequencing

Wild-type *T. trogii* S0301 was used for isolation of the homokaryotic strain, which was obtained using protoplast regeneration as described by Gao [47]. Fungi were grown on GYP medium (2% glucose, 0.5% yeast extract, 0.5% tryptone, 0.1% MgSO_4_·7H_2_O) at 28 °C [21]. In brief, 5-day mycelia of *T. trogii* S0301 were homogenized and hydrolyzed in a solution containing 2% (w/v) lysing enzymes from *Trichoderma harzianum* (Sigma-Aldrich, Darmstadt, Germany) in 0.6 M mannitol for osmotic stabilizer. Competent protoplasts were obtained by slowly draining the enzymolysis liquid through microporous filter cloth, then plated on solid regeneration medium (0.6 M mannitol, 2% glucose, 1% yeast extract, 1% tryptone and 1.5% agarose). Previous studies have shown that the clamp connection structure is a type that only occurs in heterokaryotic and none in the homokaryotic strains. The mating-type genes (b1 and b2) were different between heterokaryotic and homokaryotic strains, with homokaryotic strains having only the b1 mating-type gene [22, 47]. After 5 days of incubation at 28 °C, regenerated colonies had formed and homokaryotic strains were screened out by microscopic observation of mycelia and molecular identification of mating genes.

Filtered fungal mycelia were ground to a fine powder in liquid nitrogen and incubated in DNA or RNA extraction buffer. Total DNA and RNA were extracted using the EZgeneTM Fungal gDNA Kit (Biomiga, San Diego, USA) and the Eastep^®^ Super Total RNA Extraction Kit (Promega, Madison, USA), respectively.

In total, 20 μg of sheared DNA was used to construct a PacBio Sequel reads library with an insert size of 20 Kbp. The libraries were sequenced in 1 SMRT cell on the Sequel II platform.

The DNA and cDNA libraries (Insert size = 500 bp) were sequenced on the Illumina sequencing platform (Illumina, San Diego, CA, USA) using paired-end technology in a single run according to the manufacturer’s instructions. The DNA and RNA clean reads were used to survey and annotate the genome sequence.

### Genome assembly

Trimmomatic v0.36 was used to remove adapter sequences, ambiguous reads, low-quality bases (*Q* < 20), and short (≤ 36 bp) pair-end reads [48]. jellyfish v2.2.6 and GenomeScope v1.0 were used for genome survey (parameters: *k*-mer length = 21, max *k*-mer coverage = 1000) [49, 50]. The Sequel raw BAM files were converted to subreads in FASTA format using the PacBio SMRT software package (parameters: read score = 0.75, min sub-read length = 500).

The de novo assembly of the *T. trogii* S0301 was carried out using FALCON v0.3.0 (https://github.com/PacificBiosciences/FALCON), set input type as raw, set length_cutoff and length_cutoff_pr parameters from 1 to 10 kb, and selected the longest contig N50 size from the genome assembly results as the final consensus genome.

### Genome annotation and assessment

*Trametes trogii* S0301 genome was annotated by the FunGAP (Fungal Genome Annotation Pipeline) pipeline combining transcriptome-based, de novo, and homology-based predictions. To attain high-quality gene models, the pipeline runs multiple gene-prediction programs, including Augustus, Maker, and Braker [51]. We aligned protein sequences in Gene Ontology (GO) [52], NCBI Clusters of Orthologous Groups of Proteins (COG) [53], non-redundant database (nr), Swiss-Port and Kyoto Encyclopedia of Genes and Genomes (KEGG) [54] with BLAST and eggNOG-mapper [55].

Benchmarking Universal Single-Copy Orthologs (BUSCO) software was used to assess the completeness of genome assembly and annotation with single-copy ortholog [56]. BUSCO v2.0 was used with the latest fungi data sets (dataset: basidiomycota_odb9, 25 species of Basidiomycota, relation date: 2016-02-13) in genome mode to assess the completeness of the genome. The phylogenetic tree was constructed using the maximum likelihood method (parameters: bootstrap = 1000) in MEGA v10.0.5 by aligning the 18S ribosomal RNA (18S rRNA) gene sequences with 38 species obtained from the SILVA database [57, 58].

### Repeat annotation

RepeatMasker is a program that screens DNA sequences for interspersed repeats and low complexity DNA sequences [59]. Repeats in the *T. trogii* S0301 genome were identified using RepeatMasker v4.0.5. Scanning the *T. trogii* S0301 genome assembly for repeats as defined in the Repbase database [60].

### Ortholog clustering

Ortholog clustering and gene-family clustering analyses were performed using OrthoMCL on all protein-coding genes of *T. trogii*, *T. coccinea*, *T. versicolor*, *T. pubescens*, *Daedalea quercina*, *Ganoderma sinense*, *Grifola frondosa*, *Laetiporus sulphureus*, *Obba rivulosa*, *Phlebia centrifuga*, *Postia placenta*, and *Wolfiporia cocos*. The analyses were performed as described in the OrthoMCL manual [61].

### CAZyme genes in the *T. trogii* S0301genome

Carbohydrate-active enzymes (CAZymes) are very important to the biotech industry, particularly the emerging biofuel industry because they are responsible for the synthesis, degradation, and modification of all carbohydrates [62]. The plant cell wall-modifying and -degrading enzymes secreted by microorganisms have been classified into six classes: GHs, GTs, PLs, CEs, AAs, and CBMs [63]. CAZyme gene detection was performed using CAZyDB and HMMER v3.2 [63, 64].

### Genes from the laccase pathway in the *T. trogii* S0301 genome

The laccase pathway (EC 1.10.3.2) related genes were downloaded from the KEGG database (https://www.kegg.jp), obtaining 2404 protein sequences. We searched the CDS protein sequences against these genes by BLASTP and filtered the result using a python script (threshold value *E* ≤ 0.1, identity ≥ 30% and coverage ≥ 50%). SignalP v4.1 was used to predict signal peptides [65]. Blast2GO was used to verify and annotate the identified genes [66].

### Sample preparation for enzymatic and transcriptome assays

For enzyme assays and RNA extraction, the inocula were prepared in 250 mL Erlenmeyer flask containing 50 mL Highley’s basal salt medium [67] starting from four mycelial plugs (1 cm in diameter). Cultures were homogenized by beaded glasses (0.3 mm in diameter) after growing for 5 days at 28 °C, and 5% (v/v) aliquots of the mycelia suspension were transferred to Highley’s basal salt medium containing 1% (w/v) glucose (1% G), 0.5% (w/v) glucose and 0.5% (w/v) ball-milled oak woods (0.5% LG), and 1% (w/v) ball-milled oak woods (1% L). The Erlenmeyer flasks were incubated at 28 °C for 10 days with shaking. The mycelia and supernatants were harvested every 24 h by centrifugation at 8000*g* for 10 min. All samples were immediately frozen at − 80 °C for glucose concentration, enzyme activity assays and RNA extraction. All sample were in triplicate.

The quality control step using Trimmomatic v0.36 [48]. Clean reads were assembled into transcripts using TopHat and Cufflinks with the *T. trogii* S0301 genome as Ref. [68]. The gene expression levels were conducted using the transcripts per million (TPM) method [69].

### Enzymatic assays

The activities of cellucase, EG and xylanase were determined by 3, 5-dinitrosalicylic acid (DNS) method using filter paper, birch wood xylan or carboxymethylcellulose as the substrates [70]. One unit of enzyme activity was defined as the amount of enzyme that produced 1 μmol of reducing sugars per min.

The β-glucosidase activity was determined as previously described with some modifications [71]. The appropriately diluted crude enzyme was incubated at 50 °C in 200 mM disodium hydrogen phosphate-citric acid buffer (pH 6.0) containing 1 mM 4-nitrophenyl alpha-d-glucoside. Thirty minutes later, the reactions were stopped by adding 100 μL of 1 M Na_2_CO_3_ solution. The amount of ρ-nitrophenol (ρNP) in the reaction tubes was measured at 405 nm, and one unit of activity was defined as the amount of enzyme that released 1 µmol of ρNP per min under the test conditions.

Laccase activity was determined with 2,2′-azino-bis (3-ethylbenzothiazoline-6-sulphonic acid) (ABTS) as substrate as described by Yan et al. [70]. MnP and Lip activity was performed according to the methods of Qin et al. [43]. MnP activity was determined with MnSO_4_ as substrate in 100 mM sodium tartrate buffer (pH 5.0) containing 0.1 mM MnSO_4_ and 0.1 mM H_2_O_2_. The increase in absorbance was monitored at 238 nm for 1 min at room temperature. One unit of MnP activity was defined as the amount of enzyme that oxidized 1 μmol of Mn^2+^ per min at 25 °C. LiP activity was determined with veratryl alcohol (VA) as substrate in 50 mM sodium tartrate buffer (pH 2.5) containing 2 mM VA and 0.1 mM H_2_O_2_. One unit of LiP activity was defined as the amount of enzyme that oxidized 1 μmol of VA per min.

For each enzyme assay, control samples run in parallel containing the reaction mixture with the same amount of heat-denatured enzyme solution.

## Supplementary information

**Additional file 1.** Genome comparisons with other *Trametes* species.

**Additional file 2.** PacBio read-length distribution for *T. trogii* S0301.

**Additional file 3.** GenomeScope survey for the *T. trogii* S0301 genome.

**Additional file 4.** Protein length distributions.

**Additional file 5.** Gene annotation of the *T. trogii* S0301 genome.

**Additional file 6.** BUSCO validation of the *T. trogii* S0301 genome annotation result.

**Additional file 7.** BUSCO assessment of nine sequenced genomes of *Trametes* genus.

**Additional file 8.** Transcriptome mapping and gene annotation result of *T. trogii* S0301.

**Additional file 9.** Repeats content of the *T. trogii* S0301 genome.

**Additional file 10.** CAZymes encoded by the *T. trogii* S0301 genome.

**Additional file 11.** Genes encoding CAZymes and enzymes of the laccase pathway.

**Additional file 12.** Percentage of significantly altered CAZymes related genes family. red: up regulation; blue: down regulation.

## Data Availability

The genome sequencing data and annotation results in this paper are associated with NCBI BioProject: PRJNA480364 and BioSample: SAMN09635320. The authors state that all data necessary for confirming the conclusions presented in the article are represented fully within the article and additional files.

## Abbreviations

CYPs: cytochromes P450
CAZymes: carbohydrate-active enzymes
CDS: coding region of a gene
LTR: long terminal repeat
LINEs: long interspersed nuclear elements
SINEs: short interspersed elements
COG: Orthologous Groups of Proteins
GO: Gene Ontology
KEGG: Kyoto encyclopedia of genes and genomes
TPM: transcripts per million
GHs: glycoside hydrolases
AAs: auxiliary activities
CEs: carbohydrate esterases
GTs: glycosyl transferases
CBMs: carbohydrate-binding modules
PLs: polysaccharide lyases
LO: lignin-oxidizing enzymes
LD: lignin-degrading auxiliary enzymes
LiP: lignin peroxidase
MnP: manganese peroxidase
VP: versatile peroxidases
LPMO: lytic polysaccharide monooxygenases
GYP: glucose yeast peptone
SMRT: single molecule real-time
FASTA: text-based format for representing either nucleotide sequences or peptide sequences
BAM: binary alignment map
EG: endoglucanase
LG: glucose
DNS: 3,5-dinitrosalicylic acid
ABTS: 2,2′-azino-bis(3-ethylbenzothiazoline-6-sulphonic acid)
ρNP: ρ-nitrophenol
VA: veratryl alcohol

## References

[1] Kameshwar AKS, Qin WS (2016). Recent developments in using advanced sequencing technologies for the genomic studies of lignin and cellulose degrading microorganisms. Int J Biol Sci.

[2] Singhvi MS, Chaudhari S, Gokhale DV (2014). Lignocellulose processing: a current challenge. RSC Adv.

[3] Yang J, Li WJ, Ng TB, Deng XZ, Lin J, Ye XY (2017). Laccases: production, expression regulation, and applications in pharmaceutical biodegradation. Front Microbiol.

[4] Rivera-Hoyos CM, Morales-Alvarez ED, Poutou-Pinales RA, Pedroza-Rodriguez AM, Rodriguez-Vazquez R, Delgado-Boada JM (2013). Fungal laccases. Fungal Biol Rev.

[5] Pointing SB (2001). Feasibility of bioremediation by white-rot fungi. Appl Microbiol Biotechnol.

[6] Hilden K, Hakala TK, Lundell T (2009). Thermotolerant and thermostable laccases. Biotechnol Lett.

[7] Vasina DV, Mustafaev ON, Moiseenko KV, Sadovskaya NS, Glazunova OA, Tyurin AA (2015). The Trametes hirsuta 072 laccase multigene family: genes identification and transcriptional analysis under copper ions induction. Biochimie.

[8] Collins PJ, Dobson A (1997). Regulation of laccase gene transcription in *Trametes versicolor*. Appl Environ Microbiol.

[9] Zahmatkesh M, Spanjers H, van Lier JB (2017). Fungal treatment of humic-rich industrial wastewater: application of white rot fungi in remediation of food-processing wastewater. Environ Technol.

[10] Ortiz-Monsalve S, Dornelles J, Poll E, Ramirez-Castrillon M, Valente P, Gutterres M (2017). Biodecolourisation and biodegradation of leather dyes by a native isolate of *Trametes villosa*. Process Saf Environ.

[11] Bertrand B, Martinez-Morales F, Tinoco-Valencia R, Rojas S, Acosta-Urdapilleta L, Trejo-Hernandez MR (2015). Biochemical and molecular characterization of laccase isoforms produced by the white-rot fungus *Trametes versicolor* under submerged culture conditions. J Mol Catal B-Enzym.

[12] Ling ZR, Wang SS, Zhu MJ, Ning YJ, Wang SN, Li B, Yang AZ, Zhang GQ, Zhao XM (2015). An extracellular laccase with potent dye decolorizing ability from white rot fungus *Trametes* sp. LAC-01. Int J Biol Macromol.

[13] Baldrian P (2006). Fungal laccases—occurrence and properties. FEMS Microbiol Rev.

[14] Zheng F, An Q, Meng G, Wu XJ, Dai YC, Si J, Cui BK (2017). A novel laccase from white rot fungus *Trametes orientalis*: purification, characterization, and application. Int J Biol Macromol.

[15] Floudas D, Binder M, Riley R, Barry K, Blanchette RA, Henrissat B, Martinez AT, Otillar R, Spatafora JW, Yadav JS (2012). The paleozoic origin of enzymatic lignin decomposition reconstructed from 31 fgungal genomes. Science.

[16] Granchi Z, Peng M, Chi-A-Woeng T, de Vries RP, Hildén K, Mäkelä MR (2017). Genome sequence of the basidiomycete white-rot fungus *Trametes pubescens* FBCC735. Genome Announc.

[17] Wang JJ, Zhang YL, Xu Y, Fang W, Wang XT, Fang ZM, Xiao YZ (2015). Genome sequence of a laccase producing fungus *Trametes* sp AH28-2. J Biotechnol.

[18] Pavlov AR, Tyazhelova TV, Moiseenko KV, Vasina DV, Mosunova OV, Fedorova TV, Maloshenok LG, Landesman EO, Bruskin SA, Psurtseva NV (2015). Draft genome sequence of the fungus *Trametes hirsuta* 072. Genome Announc.

[19] Grassi E, Scodeller P, Filiel N, Carballo R, Levin L (2011). Potential of *Trametes trogii* culture fluids and its purified laccase for the decolorization of different types of recalcitrant dyes without the addition of redox mediators. Int Biodeterioration Biodegrad.

[20] Zeng XK, Cai YJ, Liao XR, Zeng XL, Li WX, Zhang DB (2011). Decolorization of synthetic dyes by crude laccase from a newly isolated *Trametes trogii* strain cultivated on solid agro-industrial residue. J Hazard Mater.

[21] Yan JP, Niu JZ, Chen DD, Chen YH, Irbis C (2014). Screening of *Trametes* strains for efficient decolorization of malachite green at high temperatures and ionic concentrations. Int Biodeterioration Biodegrad.

[22] Yan JP, Chen DD, Yang E, Niu JZ, Chen YH, Chagan I (2014). Purification and characterization of a thermotolerant laccase isoform in *Trametes trogii* strain and its potential in dye decolorization. Int Biodeterioration Biodegrad.

[23] Justo A, Hibbett DS (2011). Phylogenetic classification of *Trametes* (basidiomycota, polyporales) based on a five-marker dataset. Taxon.

[24] Madej MG, Sun LF, Yan N, Kaback HR (2014). Functional architecture of MFS d-glucose transporters. Proc Natl Acad Sci USA.

[25] Reddy CA (1995). The potential for white-rot fungi in the treatment of pollutants. Curr Opin Biotechnol.

[26] Herkommerova K, Dostal J, Pichova I (2018). Decolorization and detoxification of textile wastewaters by recombinant *Myceliophthora thermophila* and *Trametes trogii* laccases. 3 Biotech.

[27] Kamei I, Sonoki S, Haraguchi K, Kondo R (2006). Fungal bioconversion of toxic polychlorinated biphenyls by white-rot fungus, *Phlebia brevispora*. Appl Microbiol Biotechnol.

[28] Nehls U, Grunze N, Willmann M, Reich M, Kuster H (2007). Sugar for my honey: carbohydrate partitioning in ectomycorrhizal symbiosis. Phytochemistry.

[29] Chen WP, Lee MK, Jefcoate C, Kim SC, Chen FS, Yu JH (2014). Fungal cytochrome P450 Monooxygenases: their distribution, structure, functions, family expansion, and evolutionary origin. Genome Biol Evol.

[30] Rytioja J, Hilden K, Yuzon J, Hatakka A, de Vries RP, Makela MR (2014). Plant-polysaccharide-degrading enzymes from *Basidiomycetes*. Microbiol Mol Biol Rev.

[31] Kameshwar AKS, Qin WS (2018). Comparative study of genome-wide plant biomass-degrading CAZymes in white rot, brown rot and soft rot fungi. Mycology.

[32] Hakulinen N, Kiiskinen LL, Kruus K, Saloheimo M, Paananen A, Koivula A, Rouvinen J (2002). Crystal structure of a laccase from *Melanocarpus albomyces* with an intact trinuclear copper site. Nat Struct Biol.

[33] Dwivedi UN, Singh P, Pandey VP, Kumar A (2011). Structure-function relationship among bacterial, fungal and plant laccases. J Mol Catal B Enzym.

[34] Janusz G, Kucharzyk KH, Pawlik A, Staszczak M, Paszczynski AJ (2013). Fungal laccase, manganese peroxidase and lignin peroxidase: gene expression and regulation. Enzyme Microb Technol.

[35] Camarero S, Pardo I, Canas AI, Molina P, Record E, Martinez AT, Martinez MJ, Alcalde M (2012). Engineering platforms for directed evolution of laccase from *Pycnoporus cinnabarinus*. Appl Environ Microbiol.

[36] Syed K, Yadav JS (2012). P450 monooxygenases (P450ome) of the model white rot fungus *Phanerochaete chrysosporium*. Crit Rev Microbiol.

[37] Pollegioni L, Tonin F, Rosini E (2015). Lignin-degrading enzymes. FEBS J.

[38] Dashtban M, Schraft H, Syed TA, Qin W (2010). Fungal biodegradation and enzymatic modification of lignin. Int J Biochem Mol Biol.

[39] Murphy C, Powlowski J, Wu M, Butler G, Tsang A (2011). Curation of characterized glycoside hydrolases of fungal origin. Database.

[40] Lundell TK, Mäkelä MR, Hildén K (2010). Lignin-modifying enzymes in filamentous basidiomycetes—ecological, functional and phylogenetic review. J Basic Microbiol.

[41] Sánchez C (2009). Lignocellulosic residues: biodegradation and bioconversion by fungi. Biotechnol Adv.

[42] Elisashvili V, Kachlishvili E, Penninckx M (2008). Effect of growth substrate, method of fermentation, and nitrogen source on lignocellulose-degrading enzymes production by white-rot basidiomycetes. J Ind Microbiol Biotechnol.

[43] Qin X, Su X, Luo H, Ma R, Yao B, Ma F (2018). Deciphering lignocellulose deconstruction by the white rot fungus *Irpex lacteus* based on genomic and transcriptomic analyses. Biotechnol Biofuels.

[44] Duncan SM, Schilling JS (2010). Carbohydrate-hydrolyzing enzyme ratios during fungal degradation of woody and non-woody lignocellulose substrates. Enzyme Microb Technol.

[45] Ward Gary, Hadar Yitzhak, Dosoretz Carlos (2003). The Biodegradation Of Lignocellulose By White Rot Fungi. Mycology.

[46] Blanchette RA (1995). Degradation of the lignocellulose complex in wood. Can J Bot.

[47] Gao S, Zhang Y, Wu YY, Yang E, Xu HN, Chagan I, Chen YH, Yan JP (2019). A rapid method for the identification of homokaryotic strains in *Trametes trogii* S0301 and their laccase-producing ability. Chin J Appl Environ Biol.

[48] Bolger AM, Lohse M, Usadel B (2014). Trimmomatic: a flexible trimmer for Illumina sequence data. Bioinformatics.

[49] Marçais G, Kingsford C (2011). A fast, lock-free approach for efficient parallel counting of occurrences of k-mers. Bioinformatics.

[50] Vurture GW, Sedlazeck FJ, Nattestad M, Underwood CJ, Fang H, Gurtowski J, Schatz MC (2017). GenomeScope: fast reference-free genome profiling from short reads. Bioinformatics.

[51] Min B, Grigoriev IV, Choi IG (2017). FunGAP: Fungal genome annotation pipeline using evidence-based gene model evaluation. Bioinformatics.

[52] Ashburner M, Ball CA, Blake JA, Botstein D, Butler H, Cherry JM, Davis AP, Dolinski K, Dwight SS, Eppig JT (2000). Gene Ontology: tool for the unification of biology. Nat Genet.

[53] Tatusov RL, Galperin MY, Natale DA, Koonin EV (2000). The COG database: a tool for genome-scale analysis of protein functions and evolution. Nucleic Acids Res.

[54] Kanehisa M, Goto S, Hattori M, Aoki-Kinoshita KF, Itoh M, Kawashima S, Katayama T, Araki M, Hirakawa M (2006). From genomics to chemical genomics: new developments in KEGG. Nucleic Acids Res.

[55] Huerta-Cepas J, Forslund K, Coelho LP, Szklarczyk D, Jensen LJ, von Mering C, Bork P (2017). Fast genome-wide functional annotation through orthology assignment by eggNOG-Mapper. Mol Biol Evol.

[56] Waterhouse RM, Seppey M, Simao FA, Manni M, Ioannidis P, Klioutchnikov G, Kriventseva EV, Zdobnov EM (2018). BUSCO applications from quality assessments to gene prediction and phylogenomics. Mol Biol Evol.

[57] Kumar S, Stecher G, Li M, Knyaz C, Tamura KMEGAX (2018). Molecular evolutionary genetics analysis across computing platforms. Mol Biol Evol.

[58] Quast C, Pruesse E, Yilmaz P, Gerken J, Schweer T, Yarza P, Peplies J, Glöckner FO (2013). The SILVA ribosomal RNA gene database project: improved data processing and web-based tools. Nucleic acids research.

[59] Tarailo-Graovac M, Chen N (2004). Using RepeatMasker to identify repetitive elements in genomic sequences. Curr Protoc Bioinform.

[60] Jurka J, Kapitonov VV, Pavlicek A, Klonowski P, Kohany O, Walichiewicz J (2005). Repbase update, a database of eukaryotic repetitive elements. Cytogenet Genome Res.

[61] Li L, Stoeckert CJ, Roos DS (2003). OrthoMCL: identification of ortholog groups for eukaryotic genomes. Genome Res.

[62] Yin YB, Mao XZ, Yang JC, Chen X, Mao FL, Xu Y (2012). dbCAN: a web resource for automated carbohydrate-active enzyme annotation. Nucleic Acids Res.

[63] Cantarel BL, Coutinho PM, Rancurel C, Bernard T, Lombard V, Henrissat B (2009). The Carbohydrate-Active EnZymes database (CAZy): an expert resource for glycogenomics. Nucleic Acids Res.

[64] Eddy SR (2011). Accelerated profile HMM searches. Plos Comput Biol.

[65] Petersen TN, Brunak S, von Heijne G, Nielsen H (2011). SignalP 4.0: discriminating signal peptides from transmembrane regions. Nat Methods.

[66] Conesa A, Gotz S, Garcia-Gomez JM, Terol J, Talon M, Robles M (2005). Blast2GO: a universal tool for annotation, visualization and analysis in functional genomics research. Bioinformatics.

[67] Hori C, Gaskell J, Igarashi K, Kersten P, Mozuch M, Samejima M, Cullen D (2014). Temporal alterations in the secretome of the selective ligninolytic fungus *Ceriporiopsis subvermispora* during growth on aspen wood reveal this organism’s strategy for degrading lignocellulose. Appl Environ Microbiol.

[68] Trapnell C, Roberts A, Goff L, Pertea G, Kim D, Kelley DR, Pimentel H, Salzberg SL, Rinn JL, Pachter L (2012). Differential gene and transcript expression analysis of RNA-seq experiments with TopHat and Cufflinks. Nat Protoc.

[69] Mortazavi A, Williams BA, McCue K, Schaeffer L, Wold B (2008). Mapping and quantifying mammalian transcriptomes by RNA-Seq. Nat Methods.

[70] Yan J, Chen Y, Niu J, Chen D, Chagan I (2015). Laccase produced by a thermotolerant strain of *Trametes trogii* LK13. Braz J Microbiol.

[71] Zhang G, Liu P, Zhang L, Wei W, Wang X, Wei D, Wang W (2016). Bioprospecting metagenomics of a microbial community on cotton degradation: mining for new glycoside hydrolases. J Biotechnol.

