# Supplementary material for: Lignin degradation potential and draft genome sequence of Trametes trogii S0301
Source: Biotechnol Biofuels. 2019 Oct 30;12:256. doi: 10.1186/s13068-019-1596-3 (PMC6820987; doi:10.1186/s13068-019-1596-3)
Supplement: Supplementary file 1 — Additional file 1. Genome comparisons with other Trametes species. [file 13068_2019_1596_MOESM1_ESM.docx]

**Additional file 1 Genome comparisons with other *Trametes* species.**

| **Species Name** | **GeneBank**  **Number** | **GC%** | **Genome**  **Size (Mb)** | **Genes** | **Scaffolds**  **Number** | **Contig**  **Number** | **Contig N50 (bp)** |
| --- | --- | --- | --- | --- | --- | --- | --- |
| *Trametes coccinea* | PRJNA209549 | 56.6 | 32.35 | 12693 | 222 | 495 | 288560 |
| *Trametes versicolor* | PRJNA56079 | 57.7 | 44.79 | 14572 | 283 | 977 | 204373 |
| *Trametes polyzona* | PRJNA300351 | 57.4 | 36.63 | - | 5321 | 8247 | 36017 |
| *Trametes hirsuta* | PRJNA271118 | 57.6 | 37.43 | 14598 | 13 | 16 | 3045029 |
| *Trametes cinnabarina* | PRJEB5237 | 54.9 | 33.67 | 10441 | 776 | 2908 | 24093 |
| *Trametes villosa* | PRJNA435407 |  | 57.99 | - | 10327 | 1037 | 16538 |
| *Trametes pubescens* | PRJNA348811 | 57.5 | 39.74 | 14718 | 1731 | 2311 | 65985 |
| *Trametes sp. AH28-2* | PRJNA294859 | 58.8 | 38.56 | - | 306 | 502 | 307958 |
| *Trametes trogii S0301* | PRJNA480364 | 55.5 | 39.88 | 14508 | 29 | 29 | 2400359 |
|  |  |  |  |  |  |  |  |
