# Supplementary figures and images for: Lignin degradation potential and draft genome sequence of Trametes trogii S0301
Source: Biotechnol Biofuels. 2019 Oct 30;12:256. doi: 10.1186/s13068-019-1596-3 (PMC6820987; doi:10.1186/s13068-019-1596-3)

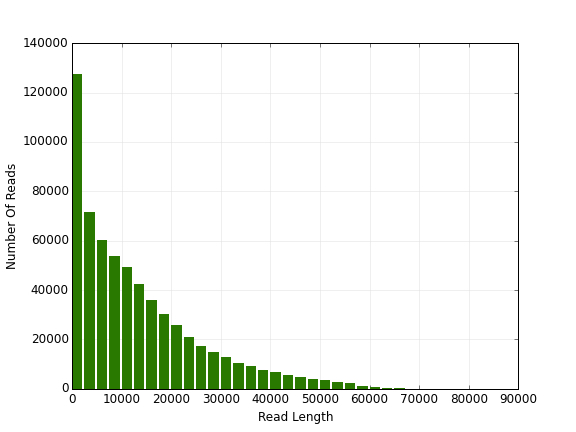

Supplement: Supplementary file 2 — Additional file 2. PacBio read-length distribution for T. trogii S0301. [file 13068_2019_1596_MOESM2_ESM.jpg]

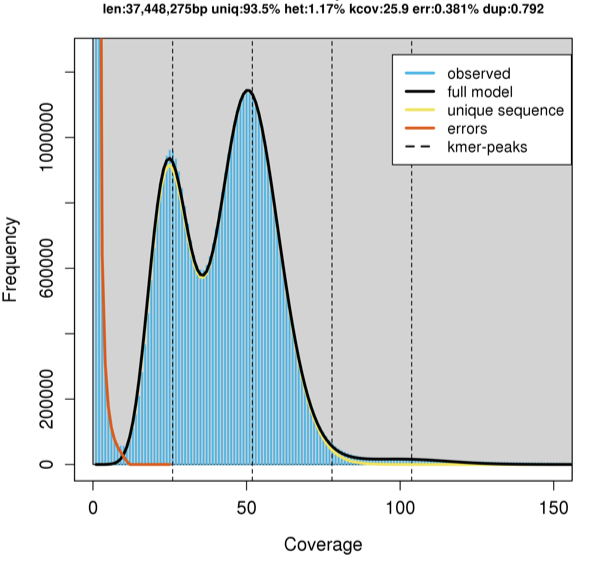

Supplement: Supplementary file 3 — Additional file 3. GenomeScope survey for the T. trogii S0301 genome. [file 13068_2019_1596_MOESM3_ESM.jpg]

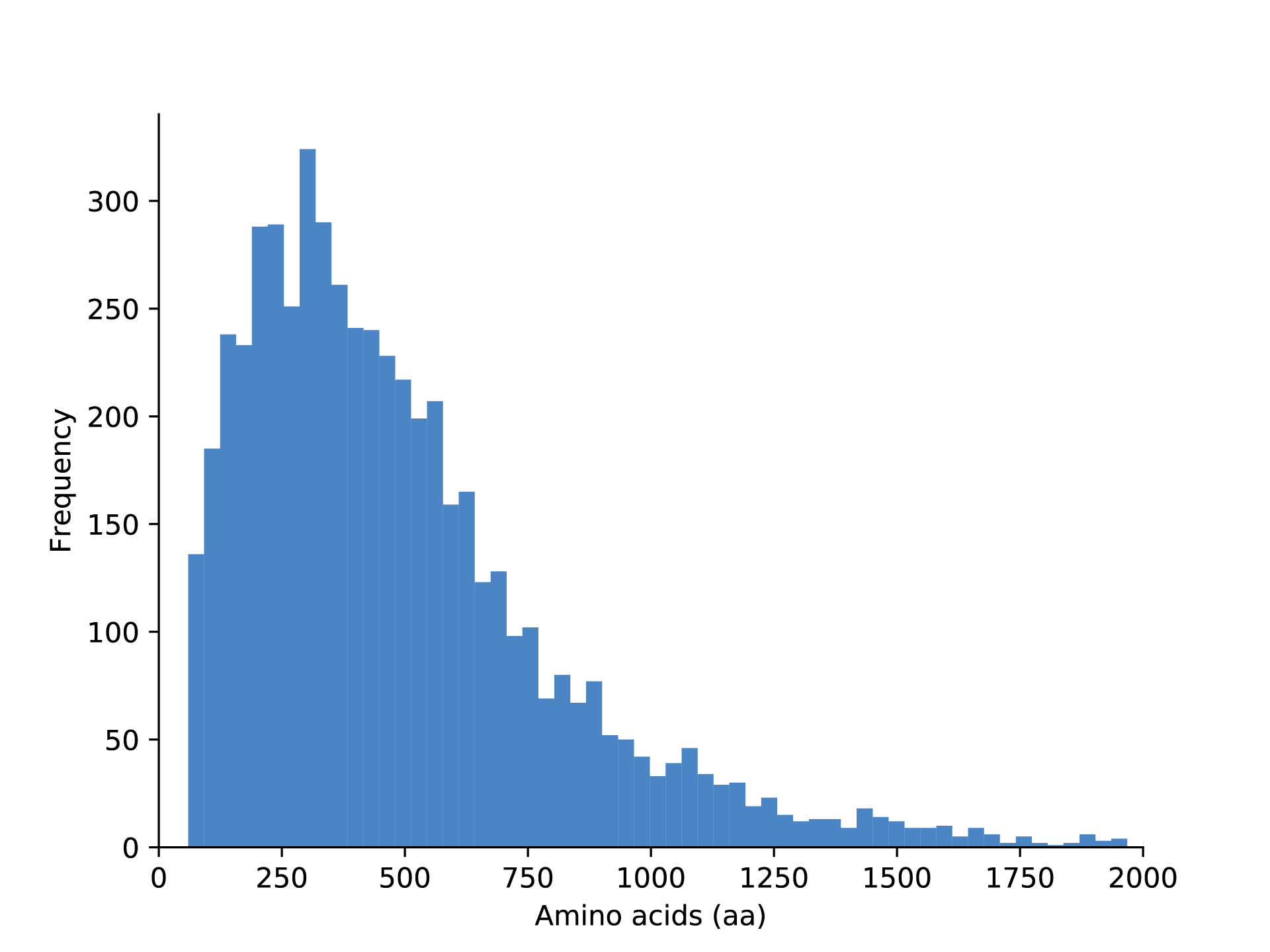

Supplement: Supplementary file 4 — Additional file 4. Protein length distributions. [file 13068_2019_1596_MOESM4_ESM.jpg]

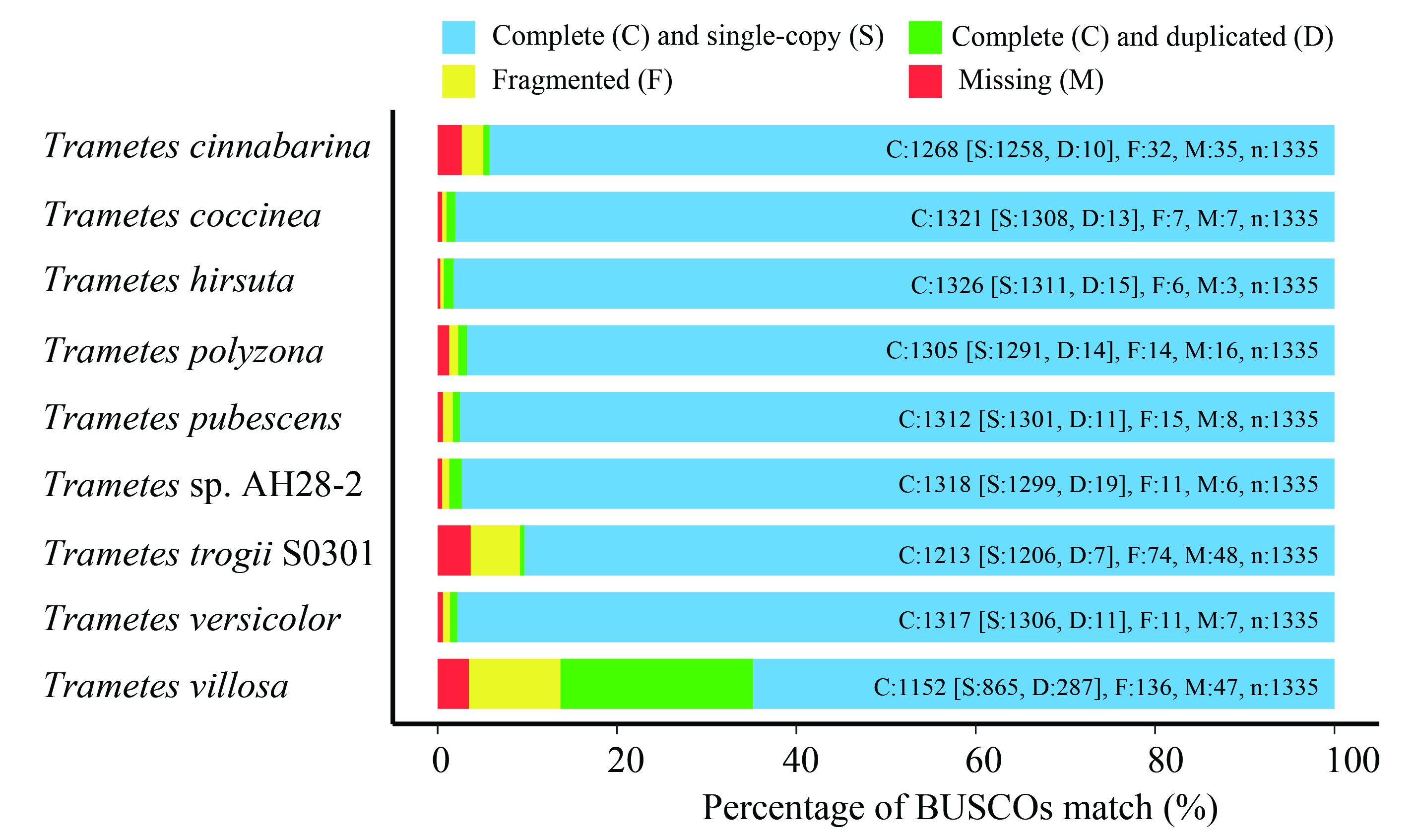

Supplement: Supplementary file 7 — Additional file 7. BUSCO assessment of nine sequenced genomes of Trametes genus. [file 13068_2019_1596_MOESM7_ESM.jpg]

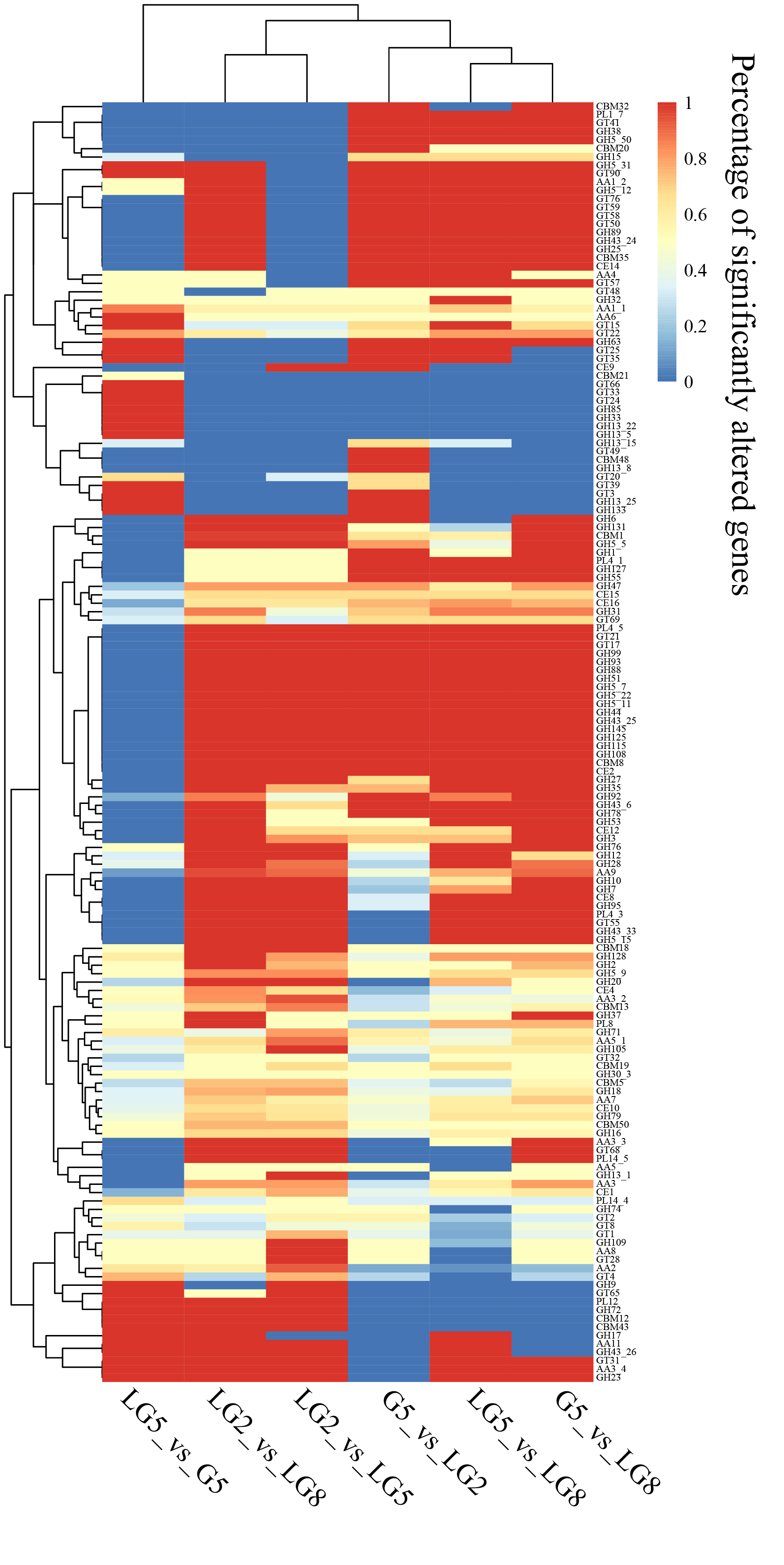

Supplement: Supplementary file 12 — Additional file 12. Percentage of significantly altered CAZymes related genes family. red: up regulation; blue: down regulation. [file 13068_2019_1596_MOESM12_ESM.jpg]
