# Supplementary material for: Lignin degradation potential and draft genome sequence of Trametes trogii S0301
Source: Biotechnol Biofuels. 2019 Oct 30;12:256. doi: 10.1186/s13068-019-1596-3 (PMC6820987; doi:10.1186/s13068-019-1596-3)
Supplement: Supplementary file 5 — Additional file 5. Gene annotation of the T. trogii S0301 genome. [file 13068_2019_1596_MOESM5_ESM.docx]

| **Attributes** | **Values** |
| --- | --- |
| **Total protein-coding genes** | 14,508 |
| **Transcript length (avg / med)** | 1,629.0 / 1,381.0 |
| **CDS length (avg / med)** | 1,257.8 / 1,044.0 |
| **Protein length (avg / med)** | 419.3 / 348.0 |
| **Exon length (avg / med)** | 211.2 / 131.0 |
| **Intron length (avg / med)** | 74.9 / 58.0 |
| **Spliced genes** | 13,377 (92.2%) |
| **Gene density (genes/Mb)** | 363.83 |
| **Number of introns** | 71,913 |
| **Number of introns per gene (med)** | 4 |
| **Number of exons** | 86,421 |
| **Number of exons per gene (med)** | 5 |

**Additional file 5 Gene annotation of the *T. trogii* S0301 genome.**
