# Supplementary material for: Lignin degradation potential and draft genome sequence of Trametes trogii S0301
Source: Biotechnol Biofuels. 2019 Oct 30;12:256. doi: 10.1186/s13068-019-1596-3 (PMC6820987; doi:10.1186/s13068-019-1596-3)
Supplement: Supplementary file 6 — Additional file 6. BUSCO validation of the T. trogii S0301 genome annotation result. [file 13068_2019_1596_MOESM6_ESM.docx]

**Additional file 6 BUSCO validation of the *T. trogii* S0301 genome annotation result.**

| **BUSCO details** | **Genome Mode** | **Proteins Mode** |
| --- | --- | --- |
| Complete BUSCOs (C) | 1213 | 1218 |
| Complete and single-copy BUSCOs (S) | 1206 | 1211 |
| Complete and duplicated BUSCOs (D) | 7 | 7 |
| Fragmented BUSCOs (F) | 74 | 84 |
| Missing BUSCOs (M) | 48 | 33 |
| Total BUSCO groups searched | 1335 | 1335 |
