# Supplementary material for: Lignin degradation potential and draft genome sequence of Trametes trogii S0301
Source: Biotechnol Biofuels. 2019 Oct 30;12:256. doi: 10.1186/s13068-019-1596-3 (PMC6820987; doi:10.1186/s13068-019-1596-3)
Supplement: Supplementary file 9 — Additional file 9. Repeats content of the T. trogii S0301 genome. [file 13068_2019_1596_MOESM9_ESM.docx]

**Additional file 9 Repeats content of the *T. trogii* S0301 genome.**

| **Type** | **Number of elements*** | **length occupied (bp)** | **percentage of sequence (%)** |
| --- | --- | --- | --- |
| **Retroelements** | 434 | 426066 | 1.07 |
| **SINEs** | 0 | 0 | 0 |
| **Penelope** | 0 | 0 | 0 |
| **LINEs** | 13 | 19497 | 0.05 |
| **LTR elements** | 421 | 406569 | 1.02 |
| **BEL/Pao** | 0 | 0 | 0 |
| **Ty1/Copia** | 193 | 129171 | 0.32 |
| **Gypsy/DIRS1** | 228 | 277398 | 0.7 |
| **Retroviral** | 0 | 0 | 0 |
| **DNA transposons** | 70 | 31148 | 0.08 |
| **hobo-Activator** | 1 | 357 | 0 |
| **Tc1-IS630-Pogo** | 34 | 10700 | 0.03 |
| **En-Spm** | 0 | 0 | 0 |
| **MuDR-IS905** | 0 | 0 | 0 |
| **PiggyBac** | 0 | 0 | 0 |
| **Tourist/Harbinger** | 10 | 5283 | 0.01 |
| **Other (Mirage, P-element, Transib)** | 2 | 107 | 0 |
| **Rolling-circles** | 0 | 0 | 0 |
| **Unclassified** | 10 | 1580 | 0 |
| **Total interspersed repeats** |  | 458794 | 1.15 |
| **Small RNA** | 26 | 28769 | 0.07 |
| **Satellites** | 0 | 0 | 0 |
| **Simple repeats** | 5767 | 262890 | 0.66 |
| **Low complexity** | 916 | 49004 | 0.12 |

* most repeats fragmented by insertions or deletions have been counted as one element. The query species was assumed to be fungi. RepeatMasker version open-4.0.5, default mode run with rmblastn version 2.2.27+.
